# The regulation of meiotic crossover distribution: a coarse solution to a century-old mystery?

**DOI:** 10.1042/BST20221329

**Published:** 2023-05-05

**Authors:** Chloe Girard, David Zwicker, Raphael Mercier

**Affiliations:** 1 Université Paris-Saclay, Commissariat à l’Énergie Atomiques et aux Énergies Alternatives (CEA), Centre National de la Recherche Scientifique (CNRS), Institute for Integrative Biology of the Cell (I2BC), Gif-sur-Yvette, France; 2 Max Planck Institute for Dynamics and Self-Organization, Am Faßberg 17, 37077 Göttingen, Germany; 3 Department of Chromosome Biology, Max Planck Institute for Plant Breeding Research, Carl-von-Linné-Weg 10, Cologne, Germany

**Keywords:** coarsening, crossover, DNA recombination, E3 ligases, HEI10, meiosis

## Abstract

Meiotic crossovers, which are exchanges of genetic material between homologous chromosomes, are more evenly and distantly spaced along chromosomes than expected by chance. This is because the occurrence of one crossover reduces the likelihood of nearby crossover events — a conserved and intriguing phenomenon called crossover interference. Although crossover interference was first described over a century ago, the mechanism allowing coordination of the fate of potential crossover sites half a chromosome away remains elusive. In this review, we discuss the recently published evidence supporting a new model for crossover patterning, coined the *coarsening model*, and point out the missing pieces that are still needed to complete this fascinating puzzle.

## Introduction

Meiotic crossovers (COs) between homologous chromosomes lead to the rearrangement of parental alleles, generating and maintaining genetic diversity. They also provide a physical link between homologs, which is required, in many species, for the correct segregation of chromosomes during the first meiotic division. The number and distribution of COs along chromosomes are neither uniform nor random and show highly conserved properties.

First, in most species, each pair of chromosomes requires at least one CO to guarantee their correct segregation at metaphase I, the so-called *obligate crossover* [1]. Second, despite a large number of DNA double-strand breaks (DSBs), which are the potential precursors of COs, only a few COs are typically formed per chromosome pair, irrespective of the size of chromosomes across species, e.g. *Plasmodium* chromosome 11 (2 Mb), canola chromosome 6 (28 Mb) and pig chromosome 6 (157 Mb) [2–6] all exhibit around three COs per meiosis. Third, COs are more distantly distributed along chromosomes than expected by chance. This is the result of a phenomenon called *crossover interference*, by which the formation of a CO at one locus interferes with and prevents the formation of another CO in its vicinity on the same chromosome. But how do potential crossover sites half a chromosome away communicate with each other to coordinate their fate and establish the final distribution? More than a century after its first detection in *Drosophila* [7–9], and its subsequent observation in a large range of species, the nature of the CO interference signal and its spreading mechanism are still the subject of lively debates [10–13].

## Crossover formation

**Figure 1.**
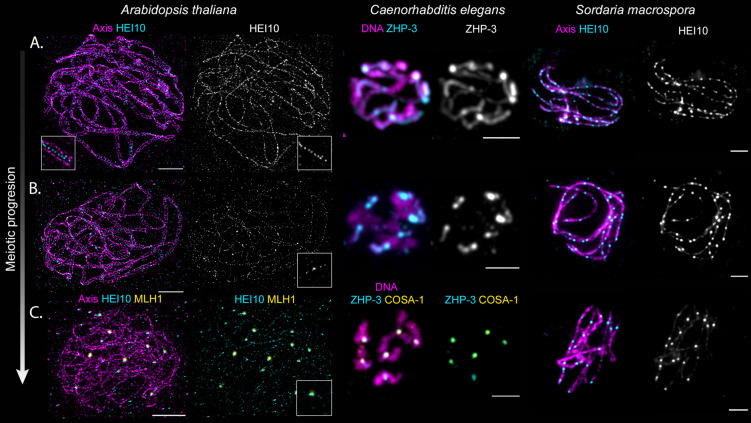
Cytological behavior of Hei10 homologs in *Arabidopsis thaliana, Caenorhabditis elegans* and *Sordaria macrospora*. (**A**) HEI10 first appears along chromosomes as multiple small foci (STED in *Arabidopsis*, in between axes marked by REC8, see inset) and/or a continuous signal (confocal microscopy in *C. elegans* and wide field microscopy in *Sordaria* with axes marked by Spo76/Pds5-TdTomato). (**B**) As meiosis progresses, bigger HEI10 foci form while others diminish in size. (**C**) Toward the end of meiotic prophase, a limited number of large HEI10 foci remain that mark CO sites, and which colocalize with MLH1 in *Arabidopsis* and COSA-1 in *C. elegans*. Scale bars 2 µm. Credits: S. Durand for *A. thaliana*, S. Köhler for *C. elegans*, C. Girard for *S. macrospora*.

Meiotic COs arise from programmed DNA DSBs and their repair by the homologous recombination machinery. Between 2- to 200-fold more DSBs than COs are formed (depending on the species, [14]): only a (small) subset will be selected to become crossovers, while the vast majority will be repaired as non-crossovers, i.e. a non-reciprocal copy of DNA sequence which does not result in chromatid exchange. In most species, two coexisting molecular pathways are responsible for crossover formation. Class I COs, which account for most events, are promoted by a group of conserved factors collectively referred to as the ZMM proteins based on *Saccharomyces cerevisiae* nomenclature (Zip1/2/3/4, Msh4/5, and Mer3). These COs can be distinguished cytologically as they are the sites of accumulation of specific pro-crossover proteins (e.g. MLH1/3 in plants and mammals, COSA-1 in *C. elegans*, Figure 1 [15–17]). Class II COs do not rely on ZMMs for their formation but on a set of endonucleases, prominently Mus81 and its partner Mms4 [18]. Class I, ZMM-dependent crossovers are sensitive to interference in all species studied thus far: they tend to be distantly spaced along chromosomes [15,19–22]. On the other hand, class II COs are not (or are much less) sensitive to interference [18,23]. They represent a minority of events in most eukaryotes. Class II COs are often considered as a backup repair mechanism for the recombination intermediates that fail to mature into class I COs or non-COs. Importantly, when the ratio between class I and class II COs is modified (e.g. in mutants that abolish class I COs, or increase class II COs), this leads to an apparent reduction in CO interference, while the process of interference itself is not modified [24,25]. This needs to be kept in mind when attributing roles in CO interference based on mutant phenotypes. In the remainder of this review, we will consider exclusively class I, ZMM-dependent, interfering crossovers.

Meiotic recombination progresses within the highly ordered environment of chromosome organization. At the onset of meiosis, chromatin is organized as an array of loops, the bases of which are tethered by proteins to form an axis [26]. The DSB machinery is assembled on this axis, and following the initiation of recombination, homologs will pair in a loose alignment all along their length [26,27]. The two axes are then ‘zipped up' to form the tripartite synaptonemal complex (SC): a transverse filament (Zip1 in *S. cerevisiae*) polymerizes progressively between the axes along the length of the homologs in a process called synapsis [26]. It is widely accepted that the mechanistic metric for interference is µm of axis/SC, rather than Mb of DNA, meaning that the interference signal propagates along the length of chromosomes axes and/or along the SC [11,27]. However, whether the tripartite SC or only the chromosome axis is required for the propagation of interference is a matter of ongoing debate ([12] and see below).

ZMM pro-crossover proteins act in concert to promote the maturation of recombination intermediates into COs. Among them, the RING-family E3 ligase HEI10–Zhp3/4–Zip3–RNF212–Vilya homologs exhibit a conserved behavior (hereafter called HEI10 for simplicity), as illustrated in Figure 1 [16,28–33]. Numerous small foci form on chromosomes as they synapse, colocalizing with the tripartite SC (Figure 1A). Super-resolution microscopy shows that HEI10 foci decorate the very middle of the SC and do not fill the axis or the entire space between the axes [29,34]. As meiosis progresses, less numerous but larger foci form (Figure 1B). This tendency for bigger foci culminates in the formation of a few intense foci corresponding to CO sites (Figure 1C). In the mouse, two RING-family E3 ligases — RNF212 and HEI10 — are essential for CO formation, and, intriguingly, RNF212 displays the cytological behavior shown by HEI10 homologs in other clades [29]. One may interpret this gradual accumulation at specific sites merely as a downstream manifestation of a CO designation decision that occurred earlier in meiotic prophase (e.g. before synapsis). In this view, HEI10 accumulation would only be the read-out of the CO designation process. Alternatively, the recently reported coarsening model proposes that this accumulation directly reflects the CO designation process itself and sees HEI10 accumulation as the driver of CO designation: only when HEI10 accumulation reaches a threshold does an embedded recombination intermediate become a CO ([22] see also below).

## What is interference, and how to measure it?

*Crossover interference* is a term that describes the tendency of crossovers to form farther away from one another than would be expected by chance. Literally, one CO *interferes* with the presence of another CO nearby on the same chromosome. This prevents the occurrence of closely spaced pairs of crossovers and generates a more even spacing of COs than would be expected if they were distributed independently from one another.

**Figure 2.**
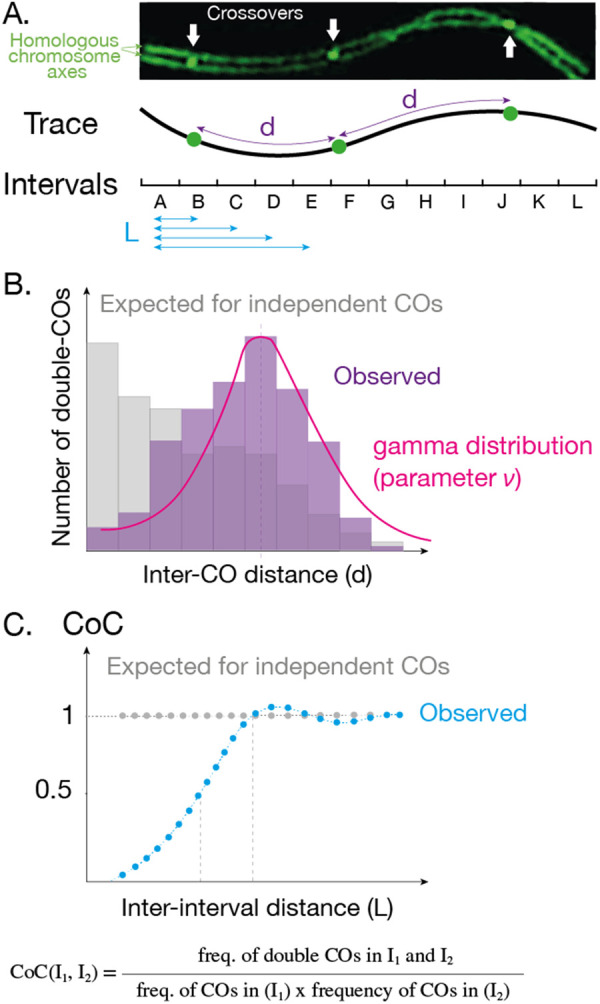
Measuring crossover interference. (**A**) There are two standard ways of measuring interference from the positions of CO events along a chromosome. Both methods can be applied on cytological (A) or genetic data. (**B**) In the first method, the distribution of distances between events (purple *d* in A) is plotted. In the presence of interference, the distribution is shifted to higher values (purple) compared with the expected in the absence of interference (gray). The parameter *ν* of the best fit *gamma* distribution is used as a measurement of interference. (**C**) In the second method, chromosomes are divided into intervals and the Coefficient of Coincidence (CoC) is calculated for all possible pairs of intervals. The CoC values are plotted against the distance between the two intervals (blue L in A). In the presence of interference, the CoC curve is close to 0 for short distances (blue dotted line), while in the absence of interference, the curve is flat at 1 (gray dotted line).

These two ways of describing the same phenomenon, (i) the larger and more even distances between neighboring COs than expected and (ii) the lack of close double COs, are reflected in two classical methods to detect and measure CO interference at the chromosome level (Figure 2). The first one examines the distribution of inter-crossover distances (Figure 2B, [35,36]): in the presence of interference, the distribution of inter-crossover distances is shifted toward larger values (purple) than if CO events were randomly distributed along the chromosome (gray). The distribution of the distances can be conveniently fitted by a gamma distribution [37], whose shape parameter *ν* gives a measurement of the strength of interference. One limitation of this approach is that the distribution can be affected by phenomena other than interference, complicating its interpretation [11,38]. The second way to measure interference is to calculate the *Coefficient of Coincidence* (CoC; Figure 2C), which effectively measures the lack of double COs compared with the expected number if COs would occur independently from each other. It compares the observed frequency of simultaneous COs in two intervals (e.g. between intervals I_1_ and I_2_, the numerator in Figure 2C), to the expected number if COs were independent (product of CO frequencies of each interval, the denominator in Figure 2C). This classic way of measuring CO interference locally [9] can be extended to an entire chromosome by calculating CoC for every pair of intervals possible. A CoC curve is obtained by plotting the CoC values against the distance *L* between the two intervals (Figure 2C). In the presence of interference, very few double crossovers are observed for close-by intervals, and the CoC is close to 0 on the left of the curve (blue in Figure 2C). For more distant intervals (longer *L*), interference vanishes, and the observed frequency of double COs approaches the expected value (CoC = 1). One possible measurement of interference strength is the length for which the value of the CoC reaches 0.5 [38]. This latter method to examine interference, which is perhaps less intuitive than the former, was shown to be more robust to other alterations of the recombination process [11,38].

These measures of interference need large datasets that can be of various kinds. One can use cytological markers of CO sites (e.g. Mlh1, COSA-1, recombination nodules) and determine their distribution along meiotic chromosomes. The metric used to describe CO distribution is, in this case, the length of axis/SC (in µm) between two adjacent events, which is likely the most relevant mechanistically [23,30,38]. COs can also be detected genetically using DNA polymorphisms between the two parents, an approach that has become more powerful with increased sequencing capability [39–43]. In this case, the distance between two adjacent COs is measured in the number of DNA base pairs. While this measurement can be very powerful, allowing high-precision mapping of CO sites and analysis of large populations, it has some limitations. First, class I and class II COs are indistinguishable by this approach, which could alter the measure of interference (see above). Second, the conversion from the DNA space in Mb to the SC space (in µm), which is relevant for mechanistic inference, may be delicate as the compaction of DNA varies along chromosomes (Table 1). Third, when sequencing gametes, only half of the events are detected, which modifies inter-CO distances [44] but not CoC curves [45].

**Table 1:** Distances at which interference acts in different species

Species	Distance of interference (µm)	Distance of interference (Mb)	Distance of interference (% of chromosome length)	Chromosome length (µm)	Chromosome length (Mb)	Chromosome compaction (Mb/µm)	Number of COs per chromosome	References
*S. cerevisiae*	0.5 µm	0.10 Mb	10–50%	1–5 µm	0.3–1.5 Mb	0.3	2–10	[38,39]
*S. macrospora*	2 µm	1.5 Mb	15–40%	5–12 µm	4–8 Mb	0.8	2–5	[30,86]
*C. elegans*	9 µm	25 Mb	120–200%	4–6 µm	14–22 Mb	3.5	1	[48,49]
*M. musculus* (male)	6 µm	110 Mb	50–180%	3–10 µm	60–195 Mb	20	1–2	[15,36,46,47]
*A. thaliana (*male)	20 µm	15 Mb	50–70%	25–40 µm	18–30 Mb	0.7	1–3	[22,70]
*S. lycopersicum*	15 µm	45 Mb	50–100%	15–30 µm	45–90 Mb	3	1–2	[23,87]

Distances are rounded approximations from experimental data hereafter. For *S. cerevisiae*: we approximated the distance at which interference has an effect from the distance at which the CoC curve of Zip3 foci reaches 1 [38] and at the peak of the distribution of inter-CO distances detected genetically [39]. For *S. macrospora*, we used the distance at which the CoC curve of HEI10 foci reaches 1 [30]. For *C. elegans*, we used the peak of the distribution of inter-CO distances (marked by COSA-1) on the *mnT12* fusion chromosome [49]. For *M. musculus*, we used the distance at which the CoC curve of Mlh1 foci [15,47] and of genetic COs [46] reaches 1. For *A. thaliana*, we used the peak of the distribution of inter-CO distances (marked by HEI10 [22]) and the distance at which the CoC curve of genetic COs reaches 1 [70]. For *S. lycopersicum*, we used the distance at which CO pairs (MLH1 foci) are as frequent as expected [23].

Intriguingly, interference acts at varying distances along chromosomes depending on the species (Table 1). In *S. cerevisiae* for instance, interference acts at a distance of roughly 0.5 µm (only 10% of the longest chromosome, [38]) while in mouse it covers most of a full chromosome [15,36,46,47]. In *C. elegans*, this distance exceeds the length of all wild-type chromosomes ensuring that one and only one CO per chromosome pair is formed [48,49]. This means that the mechanism of interference, if universal, must be able to act at both small (0.5 µm of SC) and large ranges (20 µm). A complete picture of CO patterning includes quantitative measurements of interference, CO counts and CO distribution. These different aspects of CO pattering are intertwined. Notably, CO interference influences CO counts, but also distribution. For example, if interference extends to a large proportion of the chromosome length, chromosome with exactly two COs would tend to have a CO at each end, while a CO in the middle would tend to be alone [49,50].

## The beam-film model of crossover interference

It has been widely thought that CO designation must emit a signal that propagates outward and suppresses CO formation over a certain distance on the chromosome. This led to the formulation of a compelling model in which redistribution of mechanical stress away from designated sites would prevent other COs from forming in the vicinity, a model which has come to be known as the *beam-film* model [38,51]. In physical systems, any local increase or decrease in mechanical stress at one position tends to redistribute outward from that point. In the beam-film model, the chromosomes are under mechanical stress, and at their surfaces lie an array of precursors (the DSBs). Eventually, one of these breaks reaches a threshold and undergoes a stress-promoted molecular change: CO designation. This process results in a local relaxation of stress that immediately redistributes outward from the designated event, preventing other precursors from reaching the required stress threshold to initiate CO designation in the vicinity of the first event, establishing interference. The most attractive feature of this model is that the medium for communication between COs is built into the meiotic chromosome structure: the meshwork of DNA/protein interactions at the axis should be capable of accumulating and transducing mechanical stress [52]. Supporting this model, topoisomerase II (Topo2), an enzyme able to relax over- and underwound DNA molecules [53], has been shown to be involved in modulating CO interference [38].

## The coarsening model of crossover interference

An alternative model, coined the *coarsening model*, reverses the perspective and posits that interference does not operate through the transmission of a suppressing signal but by accumulation of a pro-crossover factor at future CO sites at the expense of other neighboring ones, therefore establishing crossover interference. It was originally described, conceptualized, and supported using cytological data in *Arabidopsis* [22]. A very similar model was independently developed by another team using data from *C. elegans* [54]. This model crystallizes a corpus of previous data and ideas accumulated in the field [17,29,55].

**Figure 3.**
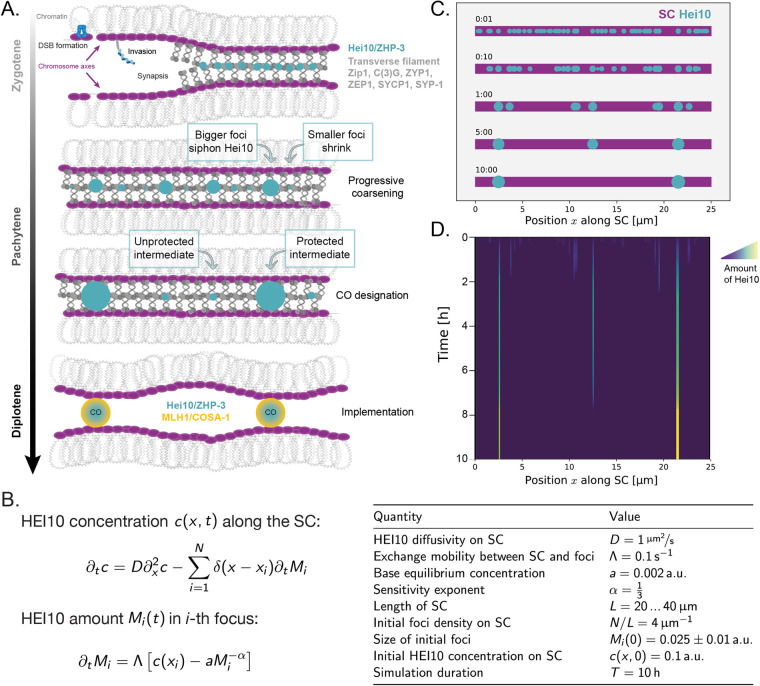
The coarsening model. (**A**) Schematic of crossover formation within the SC as envisioned by the coarsening model (**B**) A mathematical description of the coarsening model. The HEI10 concentration *c*(*x*,*t*) is defined along the entire SC of length *L*. The first equation describes its time evolution by diffusion (first term) and exchange with *N* HEI10 foci at positions *x_i_* (second term). The second equation describes the evolution of the foci sizes *M_i_*, which grow by taking up HEI10 from the SC when the equilibrium concentration a $aM_{i}^{-\alpha }$ is smaller than the concentration *c*(*x_i_*) on the SC. After initializing the system with many small foci, coarsening ensues, since larger foci exhibit a smaller equilibrium concentration, so fewer, larger foci remain after the finite simulation time *T*. (**C**) Graphical output of a simulation showing the distribution and size of HEI10 foci for one chromosome after 1 min, 10 min, 1 h, 5 h and 10 h of the coarsening process. See also Supplementary Video S1 to observe the complete process on five chromosomes of different sizes, and additional simulation replicates in Supplementary Movies S2 and S3. (**D**) Kymograph corresponding to the simulation in **C**. Shades of colors represent HEI10 density at each position. From the many foci initiated at time 0, the coarsening process yields a few large foci while the others vanish.

As mentioned above, the HEI10–Zhp3/4–Zip3–RNF212–Vilya ZMM proteins exhibit very specific dynamics during prophase, evolving from many small foci to a few large aggregates that localize at CO sites on chromosomes [28–30,32–34,56]. The coarsening model proposes that this progressive accumulation is driven by one-dimensional diffusion of HEI10 molecules along the SC. This initiates a ‘coarsening' process when bigger foci tend to capture more material than smaller foci, so larger aggregates grow at the expense of nearby smaller ones (Figure 3). As large aggregates siphon off nearby HEI10 molecules, they tend to form at a distance from one another, spontaneously creating interference. The HEI10 large foci then attract pro-CO factors that can implement the formation of a CO (resolution) at each site, such as MLH1 in plants and mammals or COSA-1 in *C. elegans* (Figures 1 and 3A). Recombination intermediates devoid of HEI10/MLH1 foci (Figure 3) would be matured into non-COs by anti-CO factors such as Sgs1(atRECQ4)-Top3-Rmi [25,57], and infrequently as class II COs. Note that this process provides an immediate explanation for the obligate crossover as, even if allowed to proceed to completion, it leads to the formation of a single large aggregate, and thus a minimum of one CO (see supplemental videos). If interrupted before completion, it leads to a limited number of aggregates that are distributed at a distance from each other. One major change of paradigm from previous models of crossover interference is that HEI10 accumulation does not only reflect the selection of the CO sites — as a readout of an upstream decision — but is the actual driving force determining CO positions.

For this model to work, two conditions must be satisfied. First, HEI10 molecules, that initially load onto the SC at multiple positions, must diffuse along the SC, but not (or at a much lower rate) between separate SCs/chromosomes (first equation in Figure 3B). The liquid-like properties of the SC may contribute to these HEI10 dynamics [54,55,58]. ZHP-3/4 has been shown experimentally in *C. elegans* to remain dynamic even after accumulation in foci [54]. The second prerequisite is that larger HEI10 aggregates should retain more HEI10 molecules than smaller aggregates (second equation in Figure 3B). The important parameters of the model that determine the eventual number of crossovers are: (i) the length *L* of the SC, (ii) the initial amount of HEI10 loaded on the SC, (iii) the diffusivity *D* of HEI10, (iv) the rate Λ of HEI10 exchange, (v) the duration *T* of coarsening (duration of pachytene), and (vi) the minimum size of HEI10 focus (threshold) that changes the fate of an underlying recombination intermediate into a CO designated site. The equations defined in Figure 3B allow us to make predictions that can be tested in different species.

This model satisfactorily accounts for a number of observations. (i) The less HEI10 foci per chromosome, the brighter each is [22]: foci on chromosomes with only one HEI10 late focus are brighter than foci on chromosomes with two or more. The coarsening model readily predicts this distinct behavior as the growth of each HEI10 focus is fueled by HEI10 proteins coming from the shrinking/disappearance of neighboring foci on the same chromosome. (ii) Intermediate Hei10 foci, which mark both sites that will and will not become crossovers, are interfering in *Sordaria* [30], which is expected if foci are growing at the expense of neighboring ones. (iii) CO count is sensitive to HEI10/RNF212 dosage in plants, mice, pig, sheep, cattle, deer and humans [29,59–70]. This could be understood in the context of the coarsening model by assuming that HEI10 dosage determines the amount of HEI10 molecules on the SC. Everything else being equal, HEI10 dosage then directly determines the CO count [22]. (iv) A strong correlation between SC length and CO number is observed. This is true within a meiocyte and between meiocytes, notably when comparing male versus female meiosis [21,71]. Assuming a fixed amount of HEI10 loaded per µm of SC, the SC length linearly determines the total amount of HEI10 introduced into the system and, consequently, CO number [70]. The SC length also influences the coarsening itself because it affects the time it takes HEI10 to diffuse along. However, this effect is likely minor compared with the effect of the total amount loaded initially. (v) The HEI10 dosage and SC length have a combined effect; increasing the HEI10 dosage increases CO proportionally to the SC length [70]. (vi) CO interference among class I COs is abolished in the absence of the transverse filament of the SC in *Arabidopsis* [34,72,73] and rice [74,75]. This is interpreted in the context of the coarsening model as follows: in the absence of the SC, diffusion of HEI10 molecules occurs within the whole nucleoplasm and these can form foci on recombination intermediates to promote CO formation throughout the nucleus. Diffusion being no longer constrained by the SC, the process is now blind to chromosomes, abolishing CO interference, the obligate CO, and the male–female CO difference that is imposed by different SC lengths [34,70,73]. (vii) Disturbing the integrity of the SC, through diminishing the amount of SC protein or removal of SC proteins, allows for more crossovers to form per chromosome [49,76,77]. This could be due to the disruption of ZHP-3 diffusion along chromosomes that would prevent the ultimate siphoning of all proteins into a single focus.

## Open questions

The coarsening model provides an intuitive basis for crossover interference, but many questions need to be addressed before it can be regarded as a convincing and comprehensive model:

- How is HEI10 diffusion constrained to the SC? The SC must have an affinity for HEI10, so as not to lose these molecules to the nucleoplasm. Multiple initial HEI10 foci are situated in the central part of the SC [29,34], suggesting a direct or indirect affinity for the N-terminus of the transverse element protein or proteins of the central element [78]. Interestingly, HEI10 and ZHP-4 RING domains (in *S. macrospora* and *C. elegans*, respectively) are required for HEI10/ZHP-3 loading on chromosomes [30,79], suggesting an important role for post-translational modifications in the loading of HEI10 on the SC. It should also be noted that HEI10 diffusion and coarsening has not yet been observed in real time *in vivo*, due to the inherent difficulty to track individual small recombination foci in a living organism for hours at a time. Leveraging the recent advances of gentle super-resolution microscopy will provide important information about HEI10 behavior during crossover formation.
- What drives coarsening? HEI10 forms foci that grow with time, suggesting that HEI10 has some effective, as yet undescribed, self-association properties. Until now, the effective coarsening of HEI10 (i.e. some foci growing while other shrink) in real time has not been observed, due to technical challenges. To trigger coarsening, larger HEI10 foci should have a stronger affinity for HEI10, thus outcompeting smaller ones. This could be explained by phase separation or, perhaps more likely, by a catalytic activity provided by HEI10 itself and/or associated proteins promoting post-translational modifications of HEI10 and/or associated proteins [54,55]. Along those lines, CDK2 phosphorylation activity in *C. elegans* is required for the aggregation of ZHP-3 [80].
- What triggers the maturation of a given HEI10 focus into a CO-designated site (i.e. MLH1/COSA-1-positive)? One possibility is the size of the focus, but the mechanism responsible remains unknown.
- What stops the coarsening process? If unstopped, the coarsening process would lead to a single focus and a single CO per chromosome (Supplementary Movies). However, 2–3 COs are typically formed per chromosome in many species. Thus, in these species, the coarsening process must be stopped before completion, presumably by triggering desynapsis and progression to the next step of meiotic prophase (diplotene). The current mathematical implementations of the model presume a fixed time, after which the coarsening is stopped. An attractive alternative possibility is the existence of a checkpoint that would trigger desynapsis, interrupting HEI10 diffusion and, therefore its coarsening, when satisfied. The checkpoint may depend on the maturation of the first HEI10 foci into CO-designated sites.
- What is the relationship between HEI10 foci and DNA recombination intermediates? For each focus to make a CO it must embed a DSB repair intermediate compatible with CO formation (a double Holliday junction). We could hypothesize that the multiple initial HEI10 foci form at DSB repair sites. This is not the most plausible model, however, as HEI10 foci outnumber the estimated numbers of DSB sites in some species [34]. Moreover, ZHP-3 and RNF212 loading onto synapsed chromosomes is independent of DSB formation [29,81] and they also load onto DNA-free poly-complexes [55,77]. Instead, the initial HEI10 loading could depend solely on the tripartite SC, and recombination intermediates would locally favor the subsequent coarsening process. RNF212 localizes to DSB repair sites in the absence of the SC, suggesting an affinity of this family of E3 ligases for recombination intermediates [29]. ZMM proteins, such as Msh4/5 or Zip2-Spo16, can bind recombination intermediates and could in turn attract HEI10 [82,83].
- What are the targets of HEI10 E3 ligase activity? And what are the roles of these targets in coarsening and recombination? Cytologically, SUMOylation of the chromosome axes is partially dependent on HEI10 and RNF212, in *Sordaria* and mouse respectively [30,84].
- Could the coarsening model coexist with other mechanisms of interference? One attractive possibility is that some mechanisms could act at relative short distances, while the coarsening mechanism would superimpose interference at longer ones. Both DSB interference [85] and stress-mediated interference [38,51] could impose a first layer of interference, preselecting recombination intermediates, among which a minority will be further selected through the coarsening process to become COs. In species with a large excess of DSBs and in which interference acts at long distances (i.e. half a chromosome, Table 1), the contribution of DSB interference is probably minor. In species with a low CO/DSB ratio [14] and/or short interference range, this contribution may be more important.

Answering these questions, which will either challenge or support the coarsening model, will require the combination of genetics, advanced microscopy, biochemistry, and modeling, which promise exciting lines of research.

## Perspectives

- Meiotic crossovers shuffle genetic information between generations in eukaryotes. Because of crossover interference, they tend to form away from each other along chromosomes through an elusive mechanism.
- An emerging model proposes that the coarsening of a conserved pro-crossover factor drives crossover distribution and interference.
- While the model accounts for numerous observations, many aspects need to be further explored.

## Open Access

Open access for this article was enabled by the participation of Max Planck Digital Library in an all-inclusive *Read & Publish* agreement with Portland Press and the Biochemical Society under a transformative agreement with MPDL.

## Abbreviations

COs: meiotic crossovers
DSBs: double-strand breaks
SC: synaptonemal complex
