# Supplementary material for: The regulation of meiotic crossover distribution: a coarse solution to a century-old mystery?
Source: Biochem Soc Trans. 2023 May 5;51(3):1179–90. doi: 10.1042/BST20221329 (PMC10317170; doi:10.1042/BST20221329)
Supplement: Supplementary Material 1 [file BST-51-1179-s1.pdf]

Supplemental videos 1-3. Numerical simulations of the coarsening model.
Five synaptonemal complexes (SCs) with lengths between $20\ \mu m$ and $40\ \mu m$ are shown as straight purple bars. Initially, foci with radii $\sim 25\ nm$ are placed at a density of four per μm SC (teal disks). These foci exchange material along the SC according to the model defined in Fig. 3B in the main text, so that only few larger foci remain after $10\ h$.
